# Metabolic Stress Impairs Pericyte Response to Optogenetic Stimulation in Pancreatic Islets

**DOI:** 10.3389/fendo.2022.918733

**Published:** 2022-06-23

**Authors:** Aurélien Michau, Chrystel Lafont, Paula Bargi-Souza, Yasmine Kemkem, Anne Guillou, Magalie A. Ravier, Gyslaine Bertrand, Annie Varrault, Tatiana Fiordelisio, David J. Hodson, Patrice Mollard, Marie Schaeffer

**Affiliations:** ^1^ Institute of Functional Genomics, Univ. Montpellier, CNRS, INSERM, Montpellier, France; ^2^ Department of Physiology and Biophysics of the Institute of Biological Sciences, Federal University of Minas Gerais (UFMG), Belo Horizonte, Brazil; ^3^ Laboratorio de Neuroendocrinología Comparada, Laboratorio Nacional de Soluciones Biomiméticas para Diagnóstico y Terapia LaNSBioDyT, Facultad de Ciencias, Universidad Nacional Autónoma de México, Mexico City, Mexico; ^4^ Oxford Centre for Diabetes, Endocrinology and Metabolism (OCDEM), National Institute for Health and Care Research (NIHR) Oxford Biomedical Research Centre, Churchill Hospital, Radcliffe Department of Medicine, University of Oxford, Oxford, United Kingdom; ^5^ Centre de Biologie Structurale, CNRS UMR 5048, INSERM U1054, Univ Montpellier, Montpellier, France

**Keywords:** diabetes, vessel, pericyte, optogenetics, pancreas, imaging, *in vivo*

## Abstract

Pancreatic islets are highly vascularized micro-organs ensuring whole body glucose homeostasis. Islet vascular cells play an integral part in sustaining adequate insulin release by beta cells. In particular, recent studies have demonstrated that islet pericytes regulate local blood flow velocity and are required for maintenance of beta cell maturity and function. In addition, increased metabolic demand accompanying obesity alters islet pericyte morphology. Here, we sought to explore the effects of metabolic stress on islet pericyte functional response to stimulation in a mouse model of type 2 diabetes, directly in the pancreas *in vivo* . We found that high fat diet induced islet pericyte hypertrophy without alterations in basal local blood flow. However, optogenetic stimulation of pericyte activity revealed impaired islet vascular responses, despite increased expression of genes encoding proteins directly or indirectly involved in cell contraction. These findings suggest that metabolic stress impinges upon islet pericyte function, which may contribute to beta cell failure during T2D.

## Introduction

The islet vasculature plays an integral role in pancreatic islet function (1). The dense, fenestrated capillary network and high blood perfusion (2) ensures adequate oxygen and nutrient supply, fast-sensing of circulating signals that regulate hormone secretion, and rapid uptake of secreted hormone into the bloodstream. In addition, islet cellular organization favours privileged interactions between beta cells and vascular cells (3). Finally, dysfunction of the islet vasculature has been associated with type 2 diabetes (T2D) pathogenesis (1).

Islet capillaries are composed of distinct cell types (1). While the role of endothelial cells in beta cell survival and function has been extensively studied (1, 4, 5), pericytes, mural cells lining capillaries, may also contribute. Pericytes regulate endothelial growth, vascular stability and blood flow in various tissues (6, 7). In islets, pericytes support beta cell maturity (8, 9) and function through platelet-derived growth factor (PDGF) signaling (10), liberation of factors that improve glucose responses (11), and enhance insulin granules exocytosis (12). In addition, islet pericytes are significant sources of islet basement membrane components, essential for beta cell function (13, 14). Finally, although different vasoactive factors, nutrients and hormones (15), including insulin (5) have long been known to regulate islet blood flow, such regulation was thought to mainly occur at the pre-capillary level (3), and a direct role for pericytes in islet blood flow regulation was recently demonstrated (16, 17), providing another level of control in addition to those of feeding arterioles. Indeed, cytosolic calcium changes in response to glucose and vasoactive molecules produced by beta cells, endothelial cells and sympathetic nerves allow islet pericytes to adjust capillary diameter (16). Since both blood flow direction and velocity within islets are important for islet function (18, 19), it was hypothesized that pericytes may contribute to glucose homeostasis through the active control of islet blood supply. Recent work using islets implanted in the anterior chamber of the eye of streptozotocin-treated diabetic mice, indeed showed that pericytes control of blood flow in islets alone is able to regulate adequate hormone secretion and systemic blood glucose levels (17).

Compelling evidence exists that vascular basement membrane thickness and fibrosis are increased (14, 20, 21), and islet blood flow is altered during conditions of impaired glucose tolerance and T2D (19, 22–24). Pericytes play a key role in peripheral diabetic vascular complications (25), and pericyte loss constitutes a very early morphological marker of diabetic retinopathy and peripheral neuropathy (26, 27). In islets, vessels are dilated and pericytes are hypertrophied in mice/rat models of insulin resistance and T2D (23, 28), and pericyte coverage decreases as T2D progresses in mice (20) and humans (16). However, it remains unknown whether pericytes may be targeted during states of metabolic stress to induce changes in islet perfusion and function. Thus, by combining high fat diet (HFD)-fed mice with *in vivo* optogenetic manipulation of pericyte activity in their native microenvironment, we provide here the first evidence that metabolic stress impinges on islet pericyte response to stimulus.

## Material and Methods

### Mice

Animal studies were conducted according to the European guidelines for animal welfare (2010/63/EU). Protocols were approved by the Institutional Animal Care and Use Committee (CEEA-LR-1191) and the French Ministry of Agriculture (APAFIS#3875). Mice were bred in specific-pathogen-free facility and housed in conventional facility during experimentation. C57BL/6J mice were purchased from Janvier-SAS (Le-Genest-St-Isle, France). NG2DsRed, NG2-Cre, and ROSA26-ChR2(H134R)-tdTomato mice (29) on a C57BL/6J background were sourced from the Jackson Laboratory (Maine, USA). To study changes in pericyte morphology following HFD-feeding, we used NG2DsRed mice, which present homogeneous labelling of both cytoplasmic and cell protrusions in NG2-positive cells, a marker commonly used to identify pericytes (30). ROSA26-ChR2-tdTomato mice were crossed to NG2-Cre mice to allow specific expression of light-sensitive ion channels in NG2-expressing cells. TdTomato labelling is however limited to the plasma membrane of NG2-positive cells in these mice, which does not allow pericyte morphological studies. Six weeks old mice were either placed on normal diet (ND) or high fat diet (HFD - 63% calories from fat) (Safe Diets) for 16 weeks. Due to protective effects of estrogens against high fat diet-induced obesity (31), only male mice were used. Intra-peritoneal glucose tolerance tests (IPGTT), insulin tolerance tests (ITT) and glucose-stimulated insulin secretion (GSIS) tests were as described (32, 33). Mice were fasted overnight before these tests.

### Manipulation of Pericyte Activity *In Vivo*

To analyze blood flow dynamics *in vivo*, the pancreas was exposed by surgery as described (33–35). Briefly, animals were anesthetized by injection of ketamine/xylazine (0.1/0.02 mg/g), placed on a heating pad, heart rate was monitored continuously and respiration was controlled by tracheotomy to limit tissue movement. The pancreas was gently maneuvered onto a metallic stage covered with a soft polymer (Bluesil) and pinned using stainless steel minutien insect pins (tip = 0.0125 mm). The tissue was continuously superfused with a NaCl 0.9% heated to 37°C. Vessels were labeled by intravenous injection of fluorescent dextran molecules (labeled with D2, 70 kDa, 25 mg/ml in NaCl 0.9%, 100 µl/20 g body weight). Fluorescence excitation was delivered by a lambda LS xenon arc lamp (300W, Sutter Instruments). Fluorescence was captured using an epi-fluorescence microscope fitted with a fast sCmos camera (ORCA Flash4.0, Hamamatsu) and a long working distance objective (2 cm, Mitutuyo, M Plan Apo ×20, NA 0.4) (36). Islet pericyte electrical activity in ROSA26-ChR2-Tomato mice crossed to NG2-Cre mice was controlled *in vivo* using computer-controlled light flashes (473 nm) (37). Laser pulse duration and power were selected so that a ~ 50% decrease in blood flow was achieved compared to basal conditions (150 ms, 1 Hz, 1400 mA). Light (473 nm Blue Solid State Laser CNI Laser, Acaltechnology) was delivered to the tissue using 200 µm diameter optic fiber (Standard Hard Cladding Multimode Fiber, Ø200 µm Core, 0.37 NA [BFL37-400], Thorlabs) placed at a maximum distance of 0.5 mm with an output power of 10mW. Controls consisted of a combination of littermate wild-type (wt), NG2-Cre and ROSA26-ChR2-Tomato mice, as results obtained in these three genotypes were not different.

### Image Data Analysis

Single Z-plane 1 min-movies were acquired. Acquisition rate was set to 150 frame/s. Blood flow in basal conditions was measured during 3.8 s before switching on the laser. A series of 10 laser flashes was then applied (150 ms flashes at a frequency of 1 Hz) and recording continued for 7 s following the last laser flash. Movies were cropped before stabilization using ImageJ. The displacement of red blood cell (RBC) shadows was visualized by contrast and in MATLAB using previously described image analysis methods (38). RBC velocities were measured before laser stimulation, during the elapsed time between the second and the third flash, the sixth and the seventh flash, the ninth and the tenth flash, and 1, 2 and 3 s after the last flash. Minimum number of frames used for RBC velocity calculation was 150. RBC velocities were measured in 5-10 vessels per movie in at least 3 different mice per condition. Changes in vessel diameter induced by optogenetic stimulation of pericytes were measured using ImageJ (line scan function). Five to ten vessels were measured prior to laser stimulation and after six flashes of laser stimulation in at least 3 independent movies from 3 different mice per condition.

### Islet Isolation and Real-Time Quantitative RT-PCR

Pancreatic islets were hand-picked after collagenase digestion of whole pancreas, as described (39). Total RNA from mouse islets was extracted using RNeasy microkit (Qiagen) following the manufacturer’s instructions. Reverse transcription was carried out using random hexamer oligonucleotides and SuperScriptIII Reverse Transcriptase (2,000U; Invitrogen, LifeTechnologies, EUA). The reverse transcription product was diluted according to the efficiency curve and submitted in duplicates to real-time quantitative PCR using LightCycler^®^ 480 SYBR Green I Master (Roche) in 7500 System (Applied Biosystems). Selection of housekeeping genes was performed using geNorm (40). PCR reactions were performed following the conditions: 95°C - 5 min, followed by 45 cycles of 95°C - 10 s and 72°C - 30 s and the Melting Curve was performed from 65 up to 97°C for 1 min. Ct values were recorded for each gene (primers listed in **Table S1**) and normalized to the geometric mean of *Ppia*, *Aldo3, Mrlp32* and *Tbp.* Ct values and then expressed relative to normal diet-fed animals (ND), according to the ddCT method (40).

### Confocal Imaging

Pancreas preparation and antibody labeling were as described (33). Images were acquired using a Zeiss LSM 780 confocal microscope. Images were analyzed using Imaris (Bitplane), Volocity (Perkin Elmer) and ImageJ (NIH). For quantifications, four slices were randomly selected from at least three animals/group, and all islets present analyzed. *A priori*, this is sufficiently-powered to detect a minimum 1.2-fold difference with a SD of 40%, a power of 0.9, and alpha = 0.05 (G*Power 3.1). Antibodies used were: rabbit anti-Ki67 (1:200, CliniSciences), guinea-pig anti-insulin (1:400, Abcam), mouse anti-glucagon (1:200, Sigma), rat anti-endomucin (1:500, Santa Cruz Biotechnology), anti-PDGFRβ (1:200, R&D Systems); alexa 647-conjugated anti-αSMA (1:200, Abcam). Nuclei were labeled using dapi (Sigma). To measure islet pericyte core area, single plane confocal images were used, and the surface occupied by a DsRed pericyte cellular body containing a nucleus was measured using ImageJ. Number of pericyte cellular bodies containing a nucleus on single plane images were counted and divided by islet surface or exocrine tissue surface in the image. The proportion of proliferative beta cells was obtained by dividing number of Ki67+ nuclei by total number of nuclei of insulin+ cells in islets, as described (33).

### Statistical Analysis

Values are represented as mean ± SEM. Statistical tests were performed using GraphPad Prism. Normality was tested using D’Agostino-Pearson test, and comparisons were made using either unpaired Student’s t-test, or two-tailed Mann-Whitney U-test, as appropriate. Multiple comparisons were made using one-way or two-way ANOVA followed by Bonferroni’s *post-hoc* test. P values were considered significant at P<0.05*, 0.01**, 0.001***, 0.0001****.

## Results

### HFD Alters Islet Pericyte Morphology and Density

**Figure 1:**
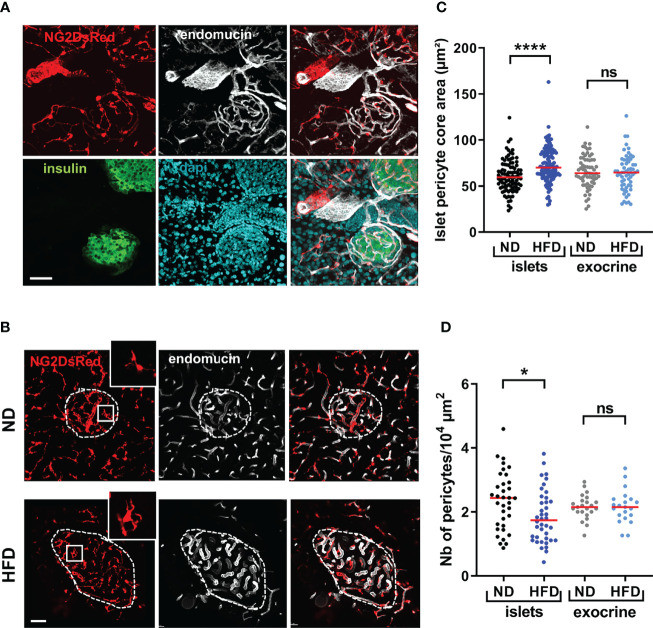
Morphological alterations of islet pericytes following 16 weeks HFD treatment. **(A)** Confocal images of pancreatic islets of a NG2DsRed mouse (scale: 50 µm, 30 µm Z-projection; red: NG2DsRed, green: insulin, white: endomucin, blue: dapi), showing NG2DsRed cellular bodies and processes line capillaries. **(B)** Representative confocal images showing pericyte hypertrophy after HFD treatment in NG2DsRed mice (scale: 50 µm, 20 µm Z-projection; red: NG2DsRed, white: endomucin). Inlet pictures correspond to enlargements of the boxed areas on left panels. ND, normal diet; HFD, high-fat diet. **(C)** Quantification of pericyte core area (n= 3-5 mice/condition, mean ± SEM, One-way ANOVA). **(D)** Number of pericyte cellular bodies containing a nucleus on single plane images were counted and divided by islet surface or exocrine tissue surface in the image (n= 3-5 mice/condition, mean ± SEM, One-way ANOVA). *P < 0.05, ****P < 0.0001. ns, non significant.

We first sought to investigate alterations in islet pericyte morphology following HFD feeding. We used mice expressing DsRed under the control of the NG2 promoter (NG2DsRed) (16, 30, 37), since NG2DsRed cells extensively cover islet capillaries (**Figure 1A**) (16, 17). We found that most NG2DsRed cells expressed the pericyte marker platelet-derived growth factor receptor beta (PDGFRβ) (**Figure S1A**) (16), and a subset of them also expressed the contractile smooth muscle actin protein (αSMA) (**Figure S1A**), consistent with previous studies that described the heterogenous nature of the pericyte population in islets (16, 17). Consistent with previous studies (32, 41–43), male mice fed HFD for 16 weeks presented with obesity, hyperglycemia, glucose intolerance, insulin resistance, defective glucose-stimulated insulin release (GSIS), and changes in islet gene expression such as *Ins1*, *Ins2* and *Gck* (**Figure S1B–H**), as expected. HFD induced alterations in pericyte morphology specifically in islets, characterized by a marked hypertrophy (**Figures 1B, C**), and a decrease in the density of pericytes per unit of islet surface (**Figure 1D**).

### Capillary Dilation Is Not Accompanied by Basal Islet Capillary Blood Flow Changes During HFD

**Figure 2:**
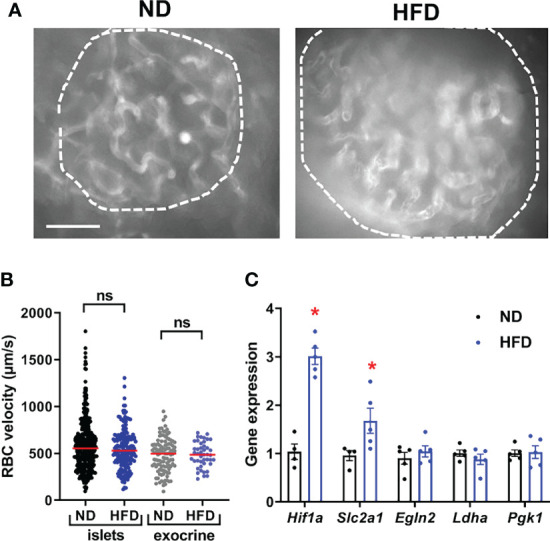
Basal islet blood flow remains unchanged following HFD despite islet hypoxia. **(A)** Still images from an *in vivo* movie of blood flow depicting an islet in a mouse fed with normal diet (ND) or high-fat diet (HFD) (See also **Video S1**) (scale: 50 µm). Vessel parenchyma was labeled with D2-dextran. Islets are circled. **(B)** Quantification of red blood cell (RBC) velocity in control (ND) and HFD-fed mice. Data is presented as mean ± SEM (n= 3-6 mice/condition, 5-10 vessels/mouse, One-way ANOVA). **(C)** Expression of hypoxia markers in islets from normal diet (ND) and high-fat diet (HFD) fed mice were measured by RT-qPCR. Data were normalized by the geometric mean of *Ppia, Aldo3, Mrlp32* and *Tbp.* Ct values and expressed as fold increase relative to ND-fed control. Data is presented as mean ± SEM (n=4-5 separate mice/group, Mann-Whitney). *P < 0.05. ns, not significant.

As expected from previous studies (42), HFD led to a marked increase in islet size and beta cell proliferation in our transgenic mouse model (**Figures S2A–C**), which was accompanied by an increase in islet capillary diameter (~35%, **Figure 2A** and **Figures S2A, D**). Consistent with the *ob/ob* mouse model (23), islet gene expression of vascular growth factor A (*Vegfa*)- an angiogenic factor abundantly expressed in islets (23) and a principal regulator of islet capillary density (44) was unchanged following HFD treatment (**Figure S2E**), suggesting vessel dilation rather than angiogenesis. Individual vessel and islet average red blood cell (RBC) velocity was similarly distributed in standard chow- and HFD-fed animals (**Figure 2B**) (**Video S1**) under conditions of clamped hyperglycaemia induced by anaesthesia [as previously described in (33)]. However, there was an increased expression of hypoxia-responsive genes in islets, such as *Hif1a* and *Glut1* (*Slc2a1*) (45) (**Figure 2C**).

### HFD Modifies Islet Pericyte Response to Optogenetic Stimulation

**Figure 3:**
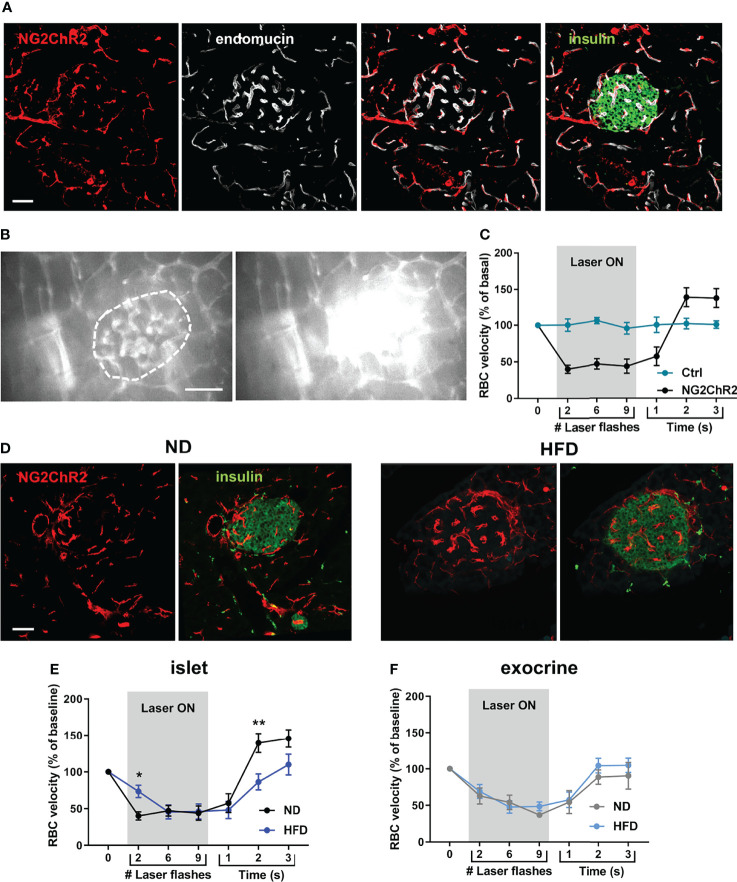
Optogenetic stimulation of islet pericytes reveals functional alteration following HFD. **(A)** Confocal images of a pancreatic islet of a NG2ChR2 mouse (scale: 50 µm, 22 µm Z-projection; red: NG2ChR2, green: insulin, white: endomucin). **(B)** Still images from an *in vivo* movie of blood flow showing blood flow alteration upon laser stimulation focused on an islet (473 nm, 150 ms, 1 Hz, 200 µm fiber at <0.5 mm of tissue) (See also **Video S2**) (scale: 50 µm). Vessel parenchyma was labeled with Rhodamine-dextran, allowing laser flashes to be clearly visible. Islet is circled. **(C)** Quantification of red blood cell (RBC) velocity in control (ChR2-Tomato or NG2-Cre mice) and NG2-ChR2 mice before, during and after laser stimulation (473 nm, 150 ms, 1 Hz, 10 flashes). Data is presented as mean ± SEM. RBC velocity is significantly reduced during laser stimulation in NG2-ChR2-Tomato mice (n= 3-6 mice/condition, 5-10 vessels/mouse, Two-way ANOVA). **(D)** Confocal images of a pancreatic islets of NG2ChR2 mice fed with normal diet (ND) (left) or high-fat diet (HFD) (right) (scale: 50 µm, 1 µm Z-projection; red: NG2ChR2, green: insulin). **(E)** Quantification of red blood cell (RBC) velocity in islets of ND-fed and HFD-fed mice before, during and after laser stimulation (473 nm, 150 ms, 1 Hz, 10 flashes). Data is presented as mean ± SEM. RBC velocity measurement shows delayed decrease and recovery of blood flow in HFD-fed animals (n= 3-6 mice/condition, 5-10 vessels/mouse, Two-way ANOVA). **(F)** Quantification of red blood cell (RBC) velocity in exocrine tissue of ND-fed and HFD-fed mice before, during and after laser stimulation (473 nm, 150 ms, 1 Hz, 10 flashes). Data is presented as mean ± SEM. RBC velocity measurement shows delayed decrease and recovery of blood flow in HFD-fed animals (n= 3-6 mice/condition, 5-10 vessels/mouse, Two-way ANOVA). *P < 0.05, **P < 0.01.

To allow the functional interrogation of pericyte activity in the intact pancreas of anesthetized mice, the blue-light sensitive cation channel ChR2 was expressed in a Cre-dependent manner using NG2-Cre animals. As expected, a similar distribution of NG2-ChR2 cells in islets was observed in NG2-Cre x ROSA26-ChR2-tdTomato mice compared to NG2DsRed animals (**Figure 3A**), and a subset of NG2-ChR2 cells expressed PBGFRβ and αSMA (**Figure S3A**). We first sought to investigate alterations in islet pericyte morphology following HFD feeding. We used mice expressing DsRed under the control of the NG2 promoter (NG2DsRed) (16, 30, 37), since NG2DsRed cells extensively cover islet capillaries (**Figure 1A**) (16, 17). We found that most NG2DsRed cells expressed the pericyte marker platelet-derived growth factor receptor beta (PDGFRβ) (**Figure S1A**) (16), and a subset of them also expressed the contractile smooth muscle actin protein (αSMA) (**Figure S1A**), consistent with previous studies that described the heterogeneous nature of the pericyte population in islets (16, 17). Male NG2-ChR2 mice fed HFD for 16 weeks presented obesity, hyperglycemia, glucose intolerance, insulin resistance, defective glucose-stimulated insulin release (GSIS), and changes in islet gene expression such as *Ins1*, *Ins2* and *Gck* (**Figure S1**). Laser stimulation was directed to islets (**Figure 3B**) or exocrine tissue areas using optic fibers (λ = 473 nm, 1 Hz, 150 ms pulses), whilst measuring blood flow, as reported (33). Consistent with a role for pericytes in islet blood flow regulation (16, 17), optical stimulation decreased RBC velocity, which could be reversed by switching OFF the laser (**Figures 3B, C**) (**Video S2**). Since we used 200 µm diameter optic fibres and NG2-positive pericytes in the exocrine tissue expressed ChR2, laser stimulation also affected RBC velocity in the exocrine tissue (**Video S2**). However, distant optogenetic stimulation in a different pancreatic lobe did not modify blood flow in the area being imaged, excluding very large scale effects (data not shown). Moreover, blood flow was not affected by optogenetic stimulation in control littermates lacking ChR2 (**Figure 3C**). Laser pulse duration and power were selected so that a ~ 50% decrease in blood flow was achieved compared to basal conditions (**Figures S3B, C**). The decrease in blood flow was associated with a decrease in capillary diameter in NG2-ChR2 mice (mean ~ 7%, corresponding to ~ 0.5 µm change in diameter) (**Figure S3D**).

Although NG2-ChR2 cells were still present in islets following HFD treatment (**Figure 3D**), their response to laser stimulation was altered (**Figure 3E**) (**Video S3**). Although laser stimulation was able to reduce RBC velocity in a reversible manner, the maximum effect was significantly delayed (**Figure 3E**). In addition, the increase in blood flow velocity observed following switching off laser stimulation was significantly less pronounced in islets from HFD treated animals (**Figure 3E**). These modifications were specific to islet pericytes, since stimulation of pericytes in the exocrine compartment yielded similar results between animals fed with normal or HFD (**Figure 3F**). This suggests a specific impairment of pericyte to optogenetic stimulation in islets following HFD treatment.

### Changes in Pericyte Marker and Contractile Protein Gene Expression in Response to HFD

**Figure 4:**
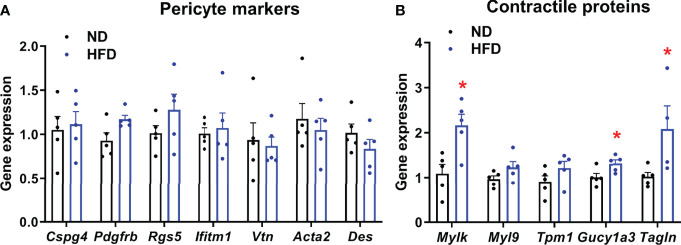
Expression of pericyte and contractile markers in islets from normal diet (ND) and high-fat diet (HFD) fed mice measured by RT-qPCR. Gene expression was measured on islets isolated from 4-5 separate mice/group. Results were normalized by the geometric mean of *Ppia, Aldo3, Mrlp32* and *Tbp.* Ct values and expressed as fold increase relative to ND-fed control. **(A)** Expression of pericyte markers in islets from normal diet (ND) and high-fat diet (HFD) fed mice measured by RT-qPCR. No significant change in expression was detected. Data is presented as mean ± SEM (n=4-5 separate mice/group, Mann-Whitney). **(B)** Gene expression profile of contractile protein markers in islets from normal diet (ND) and high-fat diet (HFD) fed mice measured by RT-qPCR. Data is presented as mean ± SEM (n = 4-5 separate mice/group, Mann-Whitney). *P < 0.05.

To further characterize the adaptive response of islets to HFD, which may contribute to functional impairment of pericytes, we analyzed changes in the expression of genes encoding pericyte markers and contractile proteins. Expression of markers of pericytes, identified previously by transcriptomic analysis of purified brain pericytes (46), was not significantly changed in islets following HFD feeding (**Figure 4A**). Since inappropriate contraction may underlie defective pericyte responses to stimulus, we analyzed expression levels of genes known to be enriched in mural cells and encoding for proteins involved in vascular smooth muscle cell contraction (46) and thus, potentially implicated in vascular dynamics. HFD increase expression of all genes analyzed, although this only reached significance for *Mylk*, *Gucy1a3* and *Tagln* (**Figure 4B**).

## Discussion

The islet vascular network provides a supportive environment for beta cells, as well as contributes to pathogenesis during T2D development (1). Based on recent findings that islet pericytes are essential for beta cell function local blood flow regulation and adequate glucose homeostasis (8, 11, 16, 17) and are morphologically altered in both human and rodents during T2D (16, 23, 28), we sought to investigate HFD-induced modifications in islet pericyte function in mice. Despite islet pericyte hypertrophy, basal local blood flow was not modified following HFD. However, islet pericyte responses to optogenetic stimulation were impaired, even in the presence of increased expression in islets of genes for proteins involved in cellular contraction. We thus provide the first evidence that HFD impinges upon islet pericyte responses in the native pancreas of anesthetized mice, which may contribute to T2D pathogenesis through inadequate adaptation of blood supply to increased metabolic demand in islets.

Pericytes are a diverse population of cells that line islet capillaries. Most of them express the NG2 and PDGFRβ markers (16), and a fraction of pericytes also express αSMA, as described (16). Of note, most αSMA pericytes are also positive for NG2 (16). Pericytes are morphologically distinguishable from arteriolar smooth muscle cells, also expressing αSMA and NG2 (37, 47, 48). In accordance with results obtained in rodent models of insulin resistance and T2D (23, 28), islet pericytes were markedly hypertrophied following 16 weeks of HFD. These morphological alterations were accompanied by a reduction of the number of pericytes per islet surface unit, consistent with islet pericyte dropout during T2D progression in humans (16). Other islet capillary changes included vessel dilation, albeit to a lesser extent than in the *ob/ob* genetic model of diabetes (~ 35% vs 128% increase in islet capillary diameter in *ob/ob* mice) (23). Nonetheless, RBC velocity in islet capillaries of HFD-fed mice was not significantly changed under basal conditions. Interestingly, there were not more stalled vessels in HFD mice arguing against a more heterogeneous constriction-dilation distribution. On the one hand, this is in accordance with measurement of islet blood flow using microspheres, which failed to detect changes in islet blood flow in islets of HFD-fed mice after correction for differences in islet volume (49). On the other hand, this contrasts with the ~ 37% increase in RBC velocity measured in islets in *ob/ob* mice (23), although this could be explained by the difference in the magnitude of vessel dilation between the two models. Indeed, the *ob/ob* model displays a profound phenotype, probably not encountered during T2D in humans, unless due to exceedingly rare leptin deficiency (50).

Notably, very small reductions in capillary diameter in tissues such as the brain induce large decrease in RBC velocity or stalling, due to the large size of RBC relative to capillary lumen (51). However, measures of changes in blood flow in relation to capillary dilation remains scarce. It is therefore difficult to predict which magnitude of increase in vessel diameter may be necessary to allow detection of flow changes, and such changes may differ from one organ to the other, reflecting different contributions of pericytes and other upstream regulation mechanisms in flow control. In addition, we previously showed that anesthesia induced marked hyperglycemia (33), which prevents measurements of RBC velocities under basal glucose levels and/or may also affect upstream mechanisms of islet blood flow regulation. Further experiments would be required to dissect the impact of islet vessel dilation on blood flow in physiological conditions.

Despite unchanged blood flow, *Hif1a* and *Glut1* (*Slc2a1*) were upregulated. Beta cell hyperplasia (52) and glucose-stimulated oxygen consumption can lead to hypoxia in beta cells from diabetic islets (53, 54). Thus, islets may become relatively hypoxic due to chronic hyperglycaemia, independently of changes in islet blood flow. In addition, perivascular accumulation of extracellular matrix, increased thickness of vascular basement membrane and fibrosis associated with T2D (20, 21, 28) may all alter nutrient and gas exchanges, although it remains unknown whether such changes occur following HFD feeding. Thus, further experiments are required to provide definitive proof of HFD-induced hypoxia in beta cells.

Contraction/relaxation of pericytes is associated with signaling factors secreted from surrounding cells (6, 16). Adenosine derived from ATP, co-secreted with insulin, has been shown to relax pericytes (16). In contrast, sympathetic nerves signals constrict islet pericytes (16). Finally, endothelial-derived vasoactive signals produced according to local oxygen levels affect pericyte activity, at least in the brain (6), and may also be involved in islets. Cell contractility is regulated by intracellular calcium levels (16), rendering pericytes as well as other NG2-expressing cells electrically-responsive to optogenetic stimulation (47, 48, 51, 55). Although a 200 µm diameter fiber optic was used to activate ChR2, we cannot exclude some off target effects on smooth muscle cells. Notably, manipulation of pericyte activity through optogenetic stimulation *in vivo* reduced local blood flow, consistent with previous reports (16, 17). This was unlikely due to local heating effects or damage, since termination of laser stimulation allowed blood flow to recover. While relatively small decreases in vessel diameter were noted, this is likely sufficient to induce large decreases in blood flow given the similar RBC and capillary lumen diameters (~ 50%). Suggesting the presence of impaired islet pericyte function during HFD, optogenetic stimulation of their activity led to delayed changes in blood flow, and impaired recovery of blood flow following termination of laser illumination. Since expression of genes encoding pericyte markers was unchanged in islets following HFD, this is unlikely to reflect a large reduction in pericyte number. Instead, this result is likely to be associated with an impaired pericyte contractile response, as HFD modified the expression of a number of genes involved in the contractile machinery (46). We cannot exclude however that changes in contractile gene expression may be linked to pericyte hypertrophy. Whether these changes are specific to islet pericytes (and not observed in acinar pericytes for example) remains to be investigated. In addition, other cells such as myofibroblasts and pancreatic stellate cells also express contractile proteins (56), and activation of these cells is known to occur in T2D (57). Going forwards scRNA-seq/snRNA-seq might be informative to dissect out the differential effects of metabolic stress on the various pancreatic pericyte populations, as well as contractile elements. Taken together, our data suggest that the adaptive response of islets to HFD fails to maintain pericyte activity. The mechanisms underlying HFD-induced changes in pericyte activity, e.g. through the study of islet pericyte responses to endogenous factors, such as adenosine and adrenergic receptor agonists following HFD, remain to be investigated.

In summary, using optogenetic activation of islet pericytes *in vivo* in the intact pancreas in mice, we show that metabolic stress impinges on islet pericyte morphology and activity. These studies thus identify a hitherto underappreciated player that may contribute to vascular complications and islet failure during T2D.

## Data Availability Statement

The original contributions presented in the study are included in the article/**Supplementary Material**. Further inquiries can be directed to the corresponding author.

## Ethics Statement

The animal study was reviewed and approved by Institutional Animal Care and Use Committee (CEEA-LR-1191) French Ministry of Agriculture (APAFIS#3875).

## Author Contributions

MS designed experiments; AM, CL, PB-S, AG, YK, MR, GB, TF, and MS performed experiments; AM, PB-S, DH, and MS analyzed data; AV provided reagents, DH, PM, and MS wrote the manuscript. All authors contributed to the article and approved the submitted version.

## Funding

Authors were supported by grants from the Agence Nationale de la Recherche (ANR BETA-DYN JCJC13 to MS, France-BioImaging ANR-10-INBS-04, ANR-15-CE14-0012 and ANR-18-CE14-0017 to PM), Société Francophone du Diabète, INSERM, CNRS, University of Montpellier, and Région Languedoc-Roussillon. DH was supported by MRC (MR/N00275X/1 and MR/S025618/1) and Diabetes UK (17/0005681) Project Grants. This project has received funding from the European Research Council (ERC) under the European Union’s Horizon 2020 research and innovation programme (Starting Grant 715884 to DH).

## Conflict of Interest

The authors declare that the research was conducted in the absence of any commercial or financial relationships that could be construed as a potential conflict of interest.

## Publisher’s Note

All claims expressed in this article are solely those of the authors and do not necessarily represent those of their affiliated organizations, or those of the publisher, the editors and the reviewers. Any product that may be evaluated in this article, or claim that may be made by its manufacturer, is not guaranteed or endorsed by the publisher.

## Abbreviations

ChR2, channelrhodopsin 2; NG2, neuroglial 2.
