# Supplementary material for: Metabolic Stress Impairs Pericyte Response to Optogenetic Stimulation in Pancreatic Islets
Source: Front Endocrinol (Lausanne). 2022 Jun 23;13:918733. doi: 10.3389/fendo.2022.918733 (PMC9259887; doi:10.3389/fendo.2022.918733)
Supplement: Supplementary file 1 [file DataSheet_1.pdf]

SUPPLEMENTAL INFORMATION

TABLE S1. List of primers used for RT-qPCR

**Symbol** GenBank#	**Primers**	**Sequences (5'- 3')**	**Amplicon length (bp)**
Aldo3	Forward	CGGCACTGGCCATATTGG	82
NM_009657.4	Reverse	ACAATAGGCACGATCCCATTCT	
Mrlp32	Forward	AGGTGCTGGGAGCTGCTACA	51
NM_029271.2	Reverse	AAAGCGACTCCAGCTCTGCT	
Tbp	Forward	ATCGAGTCCGGTAGCCGGTG	51
NM_013684.3	Reverse	GAAACCTAGCCAAACCGCC	
Ppia	Forward	CAAACACAAACGGTTCCCAG	85
NM_008907.1	Reverse	TTCACCTTCCCAAAGACCAC	
Ins1	Forward	CACTTCCTACCCCTGCTGG	81
NM_008386.3	Reverse	ACCACAAAGATGCTGTTTGACA	
Ins2	Forward	GCTTCTTCTACACACCCATGTC	147
NM_008387.3	Reverse	AGCACTGATCTACAATGCCAC	
Gck	Forward	AGGAGGCCAGTGTAAAGATGT	90
NM_010292	Reverse	CTCCCAGGTCTAAGGAGAGAAA	
Vegfa	Forward	GTGCACTGGACCCTGGCTTTA	147
NM_001025257.3	Reverse	GGTCTCAATCGGACGGCAGTA	
Hif1a	Forward	GAACATCAAGTCAGCAACGTG	150
NM_0010431.2	Reverse	TTTGACGGATGAGGAATGGG	
Slc2a1	Forward	GATTGGTTCCTTCTCTGTCGG	144
NM_011400.3	Reverse	CCCAGGATCAGCATCTCAAAG	
Egln	Forward	GCCCAGTTTGCTGACATTGAAC	184
NM_053207.2	Reverse	CCCTCACACCTTTCTCACCTGTTAG	
Ldha	Forward	ACAGTTGTTGGGGTTGGTGC	192
NM_001136069	Reverse	CGCAGTTACACAGTAGTCTTTG	
Pgk1	Forward	GATGAGGGTGGACTTCAAC	122
NM_008828.3	Reverse	TAAGGACAACGGACTTGGC	
Cspg4	Forward	CCTTCACGATCACCATCCTTC	129
NM_139001.2	Reverse	AATCATTGTCTGTTCCCCTGAG	
Pdgfrb	Forward	AGATTACGTGCCCATGTTGG	142
NM_008809	Reverse	TGGTGAGTCGTTGATTAAGGTG	
Rgs5	Forward	TTGAGTTCTGGGTTGCCTG	147
NM_009063.4	Reverse	TGGTGATGTCTTTAGTGAAGTGG	
Ifitm1	Forward	CCACAATCAACATGCCTGAG	137
NM_026820.3	Reverse	CACCATCTTCCTGTCCCTAG	
Vtn	Forward	GCTGGGAGAACATTTTCGAAC	148
NM_011707.2	Reverse	GATAAGGAGCCAGTGACGTAG	
Acta2	Forward	GTGAAGAGGAAGACAGCACAG	146
NM_007392.3	Reverse	GCCCATTCCAACCATTACTCC	
Des	Forward	CTAAAGGATGAGATGGCCCG	141
NM_010043.2	Reverse	GAAGGTCTGGATAGGAAGGTTG	
Mylk	Forward	TTGTGGCTCCTGAAGTGATC	146
NM_139300.3	Reverse	TGAAGTGACGTTGGCTAAGG	
Myl9	Forward	TTCTCTGCAGCAGGGAAACC	79
NM_172118.1	Reverse	TCTTCTTGGTGGTCTTGGCC	
Tpm1	Forward	CACATTGCTGAAGATGCTGAC	127
NM_024427	Reverse	CTTCAAGCTCGGCACATTTG	
Gucy1a3	Forward	AGTTGTCGGAGTGAAGATGC	149
NM_021896.6	Reverse	TGAACACAAAGCCAGGACAG	
Tagln	Forward	CCAGACTGTTGACCTCTATGAAG	146
NM_011526.5	Reverse	TCTTATGCTCCTGGGCTTTC	

SUPPLEMENTAL FIGURES

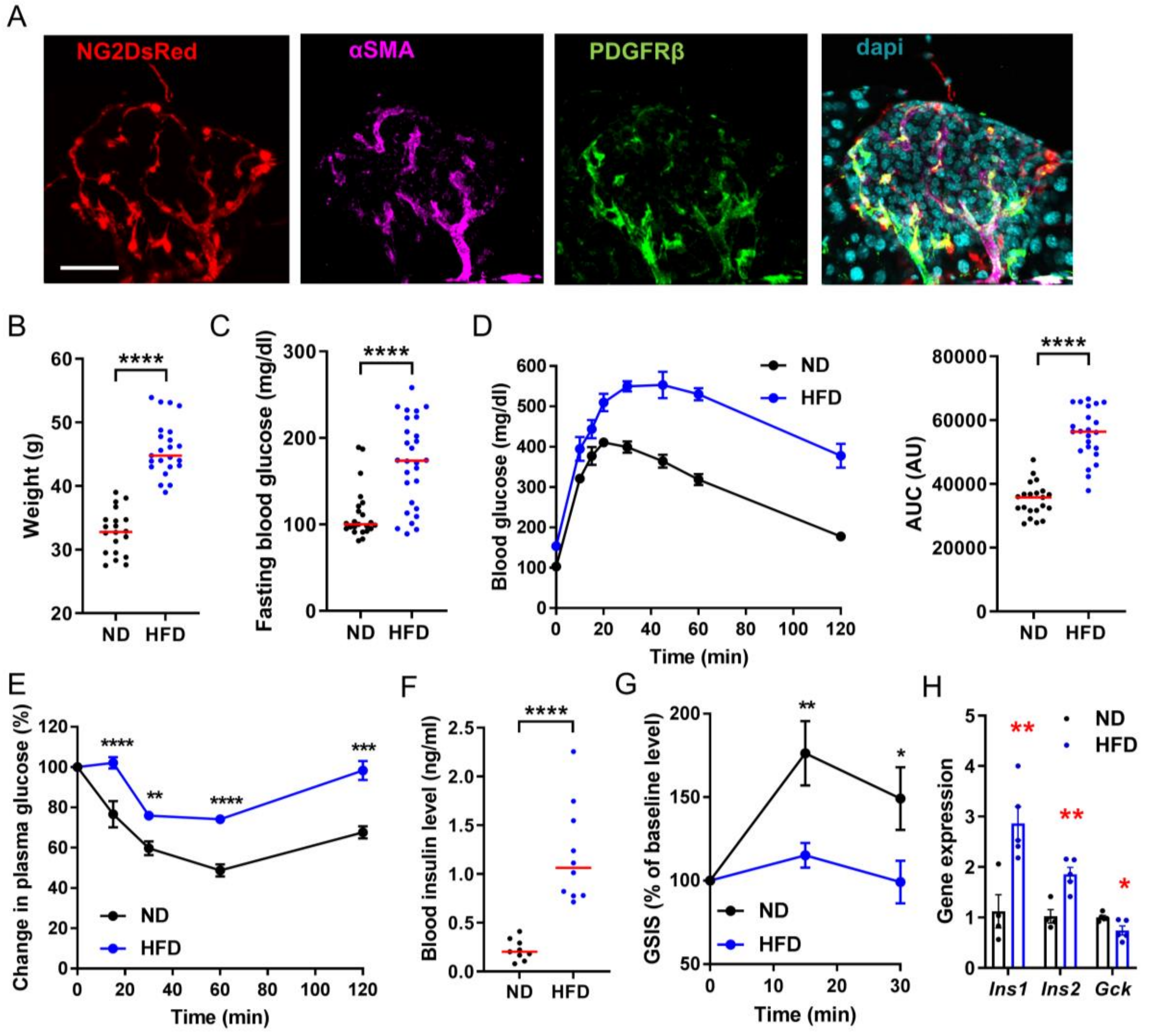

Figure S1. Pericyte labeling in islets and induction of diabetes using HFD treatment. A) Representative confocal image of an islet in a NG2DsRed mouse (scale: 50 µm, 30 µm Z-projection; red: NG2DsRed, green: PDGFRβ, purple: αSMA, blue: dapi). B-G) Feeding during 16 weeks with high-fat diet (HFD) increases body weight (B), fasting blood glucose (C), induces glucose intolerance (measured by IPGTT (3 g/kg) and area under the curve (AUC) analysis (D)) (n = 7-15 mice/group, mean ± SEM, Mann-whitney), insulin resistance (measured by ITT (0.75 UI/kg) (E)) (n = 7-9 mice/group, mean ± SEM, Two-way Anova), increased basal insulin levels (F) (n = 7-12 mice/group, mean ± SEM, Mann-whitney), and impaired glucose-stimulated insulin secretion (measured by GSIS (3 g/kg) (G)), (n = 8-9 mice/group, mean ±

SEM, Mann-Whitney). H) Expression of markers of metabolic stress in islets from normal diet (ND) and high-fat diet (HFD) fed mice were measured by RT-qPCR. The data were normalized by the geometric mean of *Ppia, Aldo3, Mrlp32* and *Tbp.* Ct values and expressed as fold increase relative to *ND*-fed control. Data is presented as mean ± SEM (n=4-5 separate mice/group, Mann-Whitney).

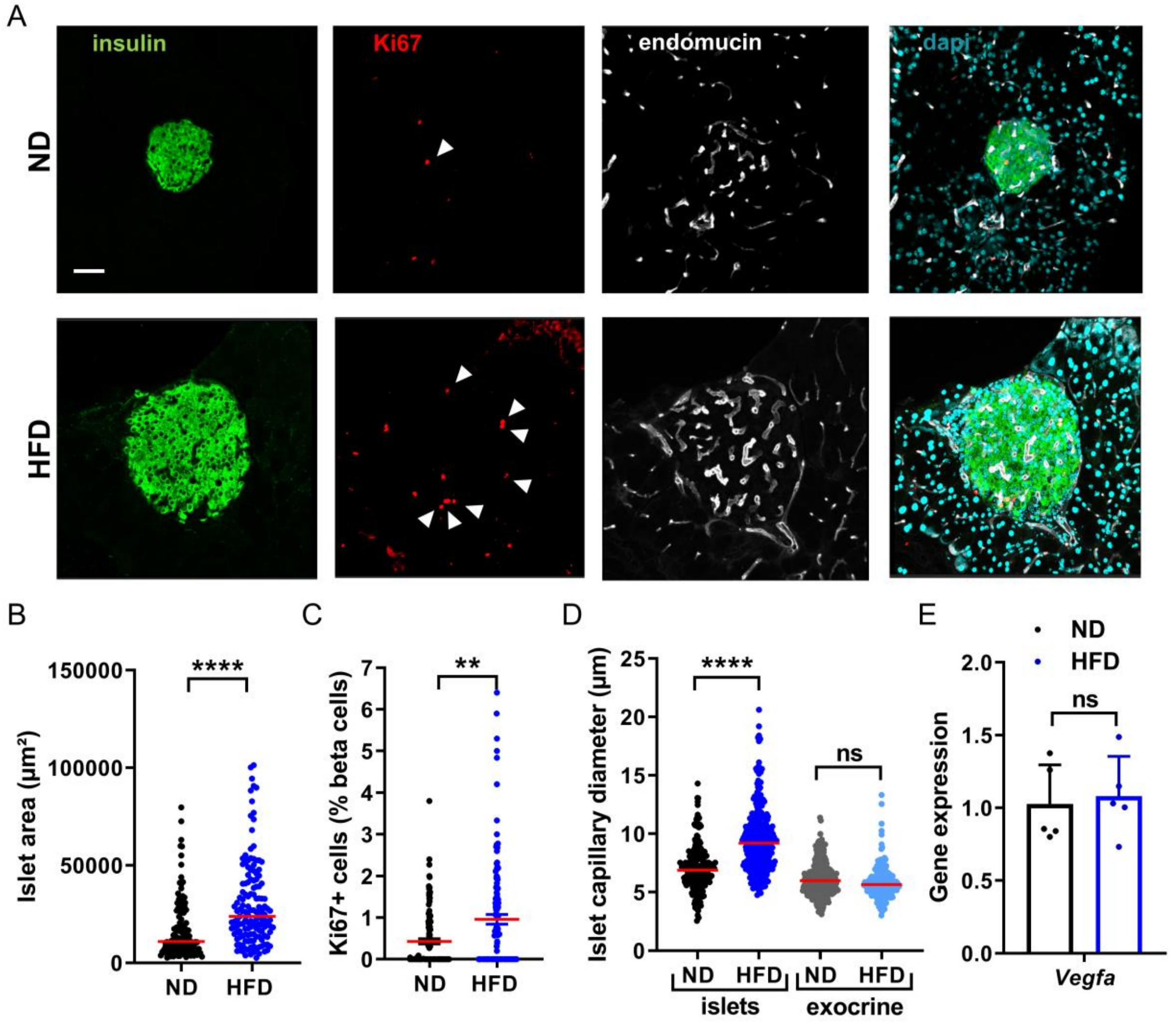

Figure S2. Effect of HFD treatment on beta cell proliferation and islet vessel morphology. A) Representative confocal images showing beta cell proliferation and vessel dilation after HFD treatment (scale: 50 µm, 10 µm Z-projection; green: insulin; red: Ki67; white: endomucin; blue: dapi). ND: normal diet; HFD: high-fat diet. B) Quantification of islet area (n = 9-12 mice/group, mean ± SEM, Mann-Whitney). C) Quantification of beta cell proliferation (measured as % of beta cells positive for Ki67) (n = 9-12 mice/group, mean ± SEM, Mann-Whitney). D) Quantification of capillary diameters in islets and exocrine tissue (n = 7-12 mice/group, mean ± SEM, One-way Anova). E) *Vegfa* expression in islets from normal diet (ND) and high-fat diet (HFD) fed mice was measured by RT-qPCR. The data were normalized by the geometric mean of *Ppia*, *Aldo3*, *Mrlp32* and *Tbp.* Ct values and expressed as fold increase relative to *ND*-fed control. Data is presented as mean ± SEM (n=4-5 separate mice/group, Mann-Whitney).

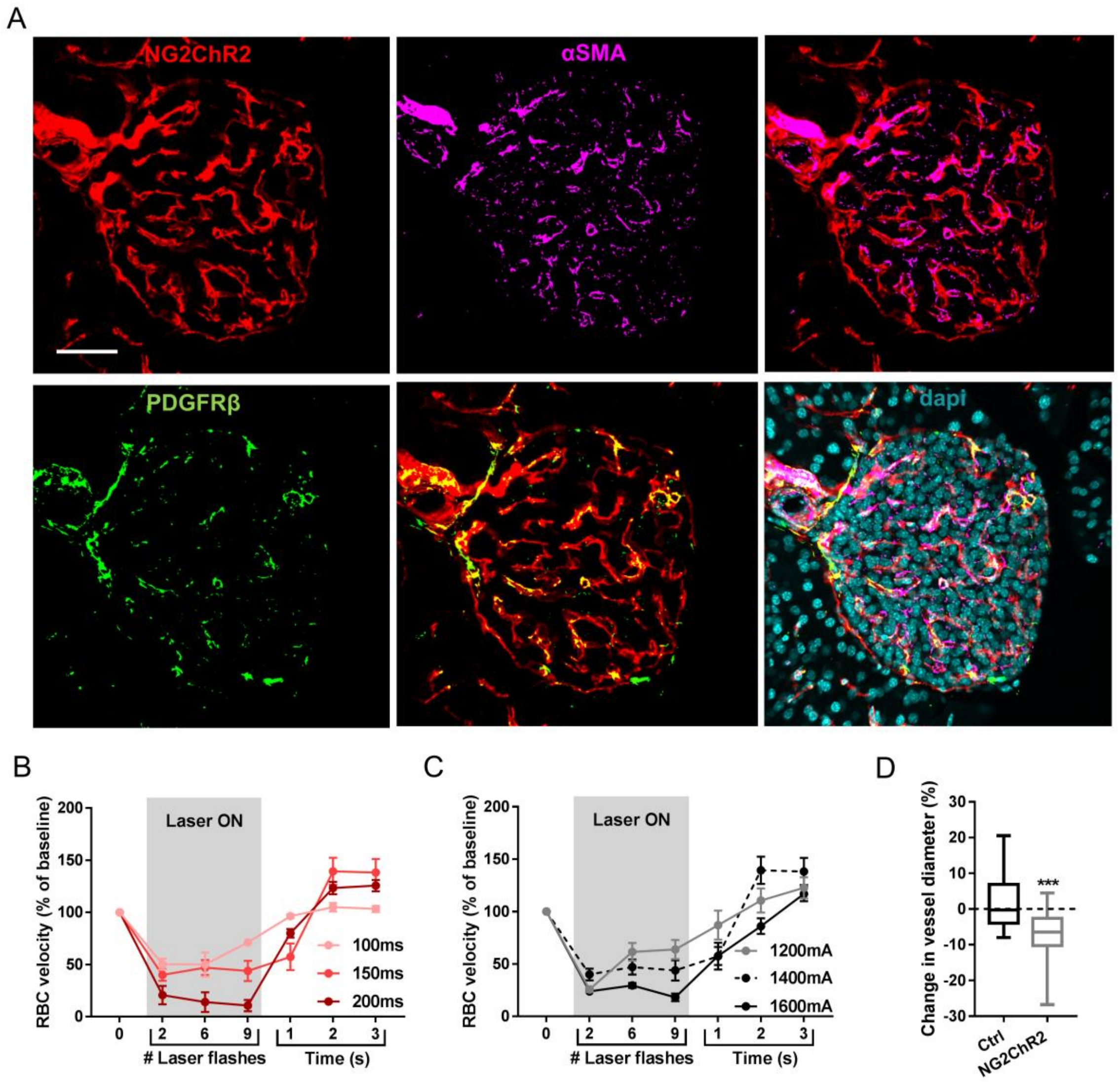

Figure S3. Optogenetic manipulation of pericytes. A) Representative confocal image of an islet in a NG2ChR2 mouse (scale: 50 µm, 20 µm Z-projection; red: NG2ChR2; green: PDGFRβ; purple: αSMA; blue: dapi). B) Quantification of red blood cell (RBC) velocity in islets of ND-fed mice before, during and after laser stimulation (473 nm, 1 Hz, 10 flashes, 10 mW output) using different laser flash durations. Data is presented as mean ± SEM (n= 3-6 mice/condition, Two-way ANOVA). C) Quantification of RBC velocity in islets of ND-fed mice before, during and after laser stimulation (473 nm, 1 Hz, 150 ms, 10 flashes) using different outputs of laser power. Data is presented as mean ± SEM (n= 3-6 mice/condition, Two-way ANOVA). D) Changes in vessel diameter expressed in percent of vessel diameter before stimulation, after 6 flashes of laser stimulation, in islets from control (ChR2-Tomato or

NG2-Cre mice) and NG2-ChR2 mice. Data is presented as mean ± SEM (n= 3-6 mice/condition, Two-way ANOVA).

SUPPLEMENTAL VIDEO LEGENDS

Video S1

Red blood cells velocity in islets *in vivo*. Representative *in vivo* recordings of blood flow following i.v. injection of 150-kDa dextran-D2, in an islet of a normal diet-fed (left) and a high-fat diet-fed mouse (right). Movie rate: 150 frames/s. Total elapsed time 3.3 s. Image size: 224×385 μm, each movie. Single z-planes. Grey: D2-labeled dextran (150 kDa).

Video S2

Optogenetic stimulation of islets. Representative *in vivo* recording of blood flow during laser stimulation (473 nm, 150 ms, 1 Hz, 10 flashes) in an islet of a normal diet-fed mouse following i.v. injection of 150-kDa dextran-rhodamine, allowing visualization of the laser-illuminated field. Movie rate: 150 frames/s. Total elapsed time 23 s. Image size: 308×564 μm. Single z-planes. Grey: 150-kDa dextran-rhodamine.

Video S3

Optogenetic stimulation of islets in normal diet and high-fat diet-fed animals. Representative *in vivo* recordings during laser stimulation (473 nm, 150 ms, 1 Hz, 10 flashes) in an islet of a normal diet-fed (left) and a high-fat diet-fed mouse (right). Flashes are faint since D2-dextran was used to label vessel parenchyma and the filter used to detect D2 filters out most of the laser signal. White bars indicate timing of laser flashes. Movie rate: 30 frames/s. Four frames out of 5 have been cut out of the original movies (original movies rate: 150 frames/s). Total elapsed time 20 s. Image size: 224×385 μm, each movie. Single z-planes.
